# A National Asian-Language Smokers’ Quitline — United States, 2012–2014

**DOI:** 10.5888/pcd12.140584

**Published:** 2015-06-25

**Authors:** Nicole Kuiper, Lei Zhang, Joann Lee, Stephen D. Babb, Christopher M. Anderson, Curt Shannon, MaryBeth Welton, Rod Lew, Shu-Hong Zhu

**Affiliations:** Author Affiliations: Lei Zhang, Stephen D. Babb, Curt Shannon, MaryBeth Welton, Centers for Disease Control and Prevention, Atlanta, Georgia; Joann Lee, Rod Lew, Asian Pacific Partners for Empowerment, Advocacy and Leadership (APPEAL), Oakland, California; Christopher M. Anderson, Shu-Hong Zhu, University of California, San Diego, California. Joann Lee was affiliated with the University of California, San Diego, during the writing of this article.

## Abstract

**Introduction:**

Until recently, in-language telephone quitline services for smokers who speak Asian languages were available only in California. In 2012, the Centers for Disease Control and Prevention (CDC) funded the national Asian Smokers’ Quitline (ASQ) to expand this service to all states. The objective of this study was to examine characteristics of ASQ callers, how they heard about the quitline, and their use of the service.

**Methods:**

Characteristics of callers from August 2012 through July 2014 were examined by using descriptive statistics. We examined demographics, cigarette smoking status, time to first cigarette, how callers heard about the quitline, and service use (receipt of counseling and medication) by using ASQ intake and administrative data. We analyzed these data by language and state.

**Results:**

In 2 years, 5,771 callers from 48 states completed intake; 31% were Chinese (Cantonese or Mandarin), 38% were Korean, and 31% were Vietnamese. More than 95% of all callers who used tobacco were current daily cigarette smokers at intake. About 87% of ASQ callers were male, 57% were aged 45 to 64 years, 48% were uninsured, and educational attainment varied. Most callers (54%) were referred by newspapers or magazines. Nearly all eligible callers (99%) received nicotine patches. About 85% of smokers enrolled in counseling; counseled smokers completed an average of 4 sessions.

**Conclusion:**

ASQ reached Chinese, Korean, and Vietnamese speakers nationwide. Callers were referred by the promotional avenues employed by ASQ, and most received services (medication, counseling, or both). State quitlines and local organizations should consider transferring callers and promoting ASQ to increase access to cessation services.

##  Introduction

More than two-thirds of smokers want to quit (1), but few use evidence-based treatments that can increase successful quitting (2,3). Telephone quitlines increase quit rates, have broad reach, and are effective with diverse populations (2). However, certain Asian subgroups experience disparities in smoking prevalence and access to cessation treatments (2,4–6). Until recently, California was the only state to provide in-language quitline services for Asian language speakers (7,8). Because some Asian subgroups with limited English fluency smoke at higher rates than those with higher English fluency (9,10), this disparity represented a gap in services in other states.

California’s experience helped address the misperception that Asians will not call quitlines because they are unfamiliar or uncomfortable with behavioral counseling (7,11). Data from 15 years of operation in California showed that Asian-language speakers were just as likely to call as English-speaking white smokers (7). A randomized controlled trial demonstrated the effectiveness of culturally tailored Asian-language protocols and services for Chinese, Korean, and Vietnamese (CKV) smokers (11). A subsequent 6-state dissemination project demonstrated the value of a multistate Asian-language quitline but suggested that a centralized promotional effort and uniform protocol for distributing nicotine replacement therapy through the quitline might be helpful (8).

In response to this evidence, in 2012, the Centers for Disease Control and Prevention (CDC) funded the Asian Smokers’ Quitline (ASQ), expanding California’s in-language program nationally. No studies to date have examined ASQ’s reach or whom it serves. CDC engaged ASQ stakeholders to design a multiyear evaluation to explore the implementation, feasibility, utility, and effectiveness of ASQ. The objective of this study is to describe the first 2 years of ASQ by examining caller characteristics, how callers heard about the quitline, and their use of the service. Results will be used to guide ASQ promotion, program improvements, and subsequent analyses.

## Methods

### Setting and sample

ASQ (www.asiansmokersquitline.org/) is operated by the University of California, San Diego (UCSD), which has also operated the California state quitline since 1992. Telephone counseling is provided by trained native speakers in Chinese (Mandarin and Cantonese), Korean, and Vietnamese. There are 3 unique telephone numbers, one for each language. Printed materials are provided in Chinese (traditional and simplified), Korean, and Vietnamese.

During the 2-year study period of August 1, 2012, to July 31, 2014, a culturally appropriate counseling protocol was delivered in each language; its development is described elsewhere (11,12). Those who called for others (proxy callers) were sent materials designed to help them support the tobacco user who wished to quit. Tobacco users who called for themselves were offered in-language self-help materials and up to 5 counseling calls. If they reported no contraindications (or, if they did, pending medical authorization), they were also offered a free 2-week supply of nicotine patches.

To promote the service, UCSD worked with an Asian-language media agency to conduct a print, digital, and radio advertising campaign in large and medium CKV media markets throughout the United States. With other community partners, it conducted a public relations campaign and outreach to community-based organizations and to physicians who serve Asian communities. CDC also ran CKV-language advertisements promoting ASQ in conjunction with the *Tips From Former Smokers* national tobacco education campaign (13); these advertisements were staggered with UCSD’s advertisements on a biweekly basis during this campaign. State tobacco control programs were invited to supplement these promotions for their own Asian-language populations by placing additional advertisements and transferring callers to ASQ.

### Data source and measures

The study included all unique callers from 3 language groups — Chinese (Cantonese and Mandarin), Korean, and Vietnamese — who completed intake from August 1, 2012, to July 31, 2014. We examined caller characteristics, how callers heard about the quitline, and services received by tobacco users. The following intake data were examined: language spoken, state of residence, sex, age, education, health insurance status, nationality/country of birth, length of time in the United States, tobacco use status, time from waking to smoking the first cigarette (ie, an indicator of nicotine dependence), and how callers heard about the quitline. Finally, we examined the proportion of tobacco users who enrolled in quitline services. For callers who received quitline services, we used ASQ administrative data to obtain the number of counseling sessions completed and whether participants received nicotine patches. The UCSD Human Research Protections Program approved the collection and use of data.

To estimate ASQ reach in California (the only state with smoking prevalence rates for CKV-language adult smokers), we calculated the ratio of California tobacco users who called ASQ for help with quitting to the total number of CKV adult smokers in California by using 2011 California Health Interview Survey data, the latest year for which public data were available (14).

Call volume was assessed and mapped by state. Descriptive frequencies for caller characteristics, how callers heard about the quitline, and their use of the service were analyzed by language group. We computed 95% confidence intervals (CIs) to facilitate comparison of caller characteristics both within and across the different language groups. Given the descriptive nature of this study, we did not correct α levels for multiple tests. SAS software version 9.3 (SAS Institute Inc) was used for all analyses.

## Results

### Call volume

During the study period, a total of 5,771 callers completed intake. Of these, 5,437 were tobacco users calling for help to quit. Calls originated from 48 of 50 states and the District of Columbia; 53% of calls came from states other than California. Vermont and Wyoming were the only states that did not have any callers. Approximately 90% of the calls came from 10 states: California (n = 2,735), New York (n = 736), Texas (n = 430), Washington (n = 312), Virginia (n = 234), Georgia (n = 204), New Jersey (n = 180), Maryland (n = 172), Illinois (n = 119), and Arizona (n = 70) (Figure). The 2,597 adult smokers in California who called ASQ over the 2-year period represent an annual reach of 1.2% of the estimated 106,924 CKV-language adult smokers in that state.

**Figure:**
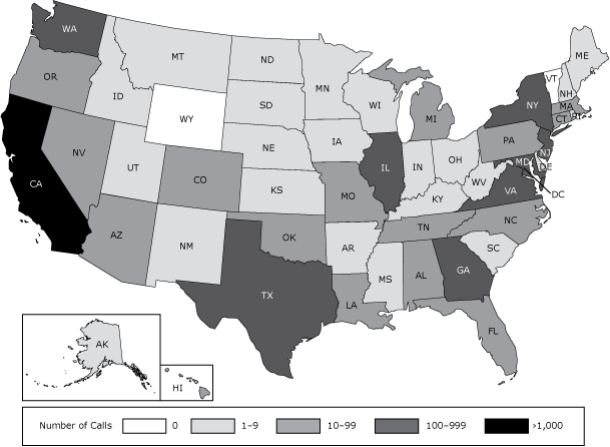
Number of calls to the Asian Smokers’ Quitline (ASQ), by state, from August 1, 2012, to July 31, 2014.

Overall, about 31% of callers (n = 1,780; 95% CI, 29.7–32.0) were Chinese, 38% (n = 2,202; 95% CI, 36.9–39.4) were Korean, and 31% (n = 1,789; 95% CI, 29.8–32.2) were Vietnamese. Ninety-five percent of all callers were tobacco users calling for themselves, and 5% were proxy callers calling on behalf of a tobacco user. Most proxy callers (84%) were female. The remaining results are presented for tobacco users who called for themselves for assistance with quitting.

### General demographics and tobacco user characteristics

 Nearly all callers (>99%) were foreign born, although there were differences between groups in the mean number of years in the United States (23 years for Koreans; 20 years for Vietnamese; and 16 years for Chinese) (Table 1). Most of these callers were male, although males were a higher proportion of Vietnamese callers (94%) than Chinese (86%) or Korean (82%) callers. Most callers (57%) were aged 45 to 64, and 25% were aged 25 to 44. The proportion of callers aged 25 to 44 was higher among Chinese (36%) callers than among Vietnamese (23%) or Koreans (19%). A higher proportion of Koreans (62%) and Vietnamese (60%) than Chinese (49%) were in the 45 to 64 age group.

**Table 1:** Characteristics of Tobacco Users Participating in the Asian Smokers’ Quitline, by Language, August 1, 2012–July 31, 2014

Characteristics	Chinese^a^	Korean^a^	Vietnamese^a^	Total, % (n)
	% (95% CI) [n]			
**Sex**				
Male	86.3 (84.6–88.0) [1,410]	82.2 (80.5–83.8) [1,737]	94.0 (92.8–95.1) [1,587]	87.1 (4,734)
Female	13.7 (12.0–15.4) [224]	17.8 (16.2–19.5) [377]	6.0 (4.9–7.2) [102]	12.9 (703)
**Age, y**				
<18	0.2 (0–0.4) [3]	0 [0]	0 [0]	0.1 (3)
18–24	2.1 (1.4–2.8) [35]	0.7 (0.4–1.1) [15]	1.3 (0.8–1.9) [23]	1.3 (73)
25–44	35.6 (33.2–37.9) [582]	18.8 (17.1–20.4) [397]	23.2 (21.2–25.2) [397]	25.2 (1,376)
45–64	49.4 (46.9–51.8) [808]	61.5 (59.5–63.6) [1,303]	59.7 (57.3–62.0) [1,021]	57.3 (3,132)
>65	12.8 (11.2–14.4) [209]	19.0 (17.3–20.7) [402]	15.8 (14.1–17.5) [270]	16.1 (881)
**Education**				
Less than high school graduation and no GED	36.0 (33.6–38.4) [560]	5.6 (4.6–6.6) [113]	39.5 (37.1–41.9) [648]	25.4 (1,321)
High school or GED	28.4 (26.2–30.7) [442]	30.4 (28.3–32.4) [611]	27.4 (25.2–29.5) [449]	28.8 (1,502)
Some college or trade school, or an associate’s degree	10.4 (8.9–11.9) [162]	15.1 (13.5–16.7) [304]	23.4 (21.4–25.5) [384]	16.3 (850)
Bachelor’s degree	21.9 (19.9–24.0) [341]	43.0 (40.8–45.1) [865]	9.0 (7.6–10.4) [148]	26.0 (1,354)
Postgraduate degree	3.2 (2.3–4.0) [49]	6.0 (4.9–7.0) [120]	0.7 (0.3–1.1) [11]	3.5 (180)
**Health insurance status**				
Private	20.7 (18.7–22.7) [328]	20.6 (18.8–22.3) [422]	27.3 (25.2–29.5) [449]	22.7 (1,199)
Public	28.3 (26.1–30.5) [447]	24.0 (22.1–25.8) [491]	36.3 (34.0–38.6) [597]	29.1 (1,535)
None	51.0 (48.5–53.4) [806]	55.4 (53.3–57.6) [1,136]	36.4 (34.0–38.7) [598]	48.2 (2,540)
**Mean no. of years in United States**	16.4 (15.8–16.9) [1,641]	22.8 (22.3–23.3) [2,117]	20.0 (19.5–20.5) [1,711]	20.0 (5,469)
**Cigarette smoking status at intake**				
Daily	94.8 (93.7–95.9) [1,556]	95.7 (94.8–96.6) [2,026]	96.3 (95.4–97.2) [1,648]	95.6 (5,230)
Some days	1.7 (1.1–2.3) [28]	0.4 (0.1–0.7) [9]	0.6 (0.2–0.9) [10]	0.9 (47)
Already quit	3.4 (2.5–4.2) [55]	3.6 (2.8–4.4) [76]	2.9 (2.1–3.7) [50]	3.3 (181)
Use of noncigarette tobacco product	0.1 (0–0.3) [2]	0.3 (0.1–0.5) [6]	0.2 (0–0.4) [3]	0.2 (11)
**Time to first cigarette upon waking is ≤5 min^b^**	37.7 (35.3–40.1) [583]	38.5 (36.4–40.6) [784]	30.2 (27.9–32.4) [490]	35.7 (1,857)

Abbreviations: CI, confidence interval; GED, graduate equivalency degree.

a All values are percentage (95% confidence interval) [N], unless otherwise indicated.

b Time to first cigarette (≤5 min) was defined as the proportion of current cigarette smokers whose response to “How soon after you wake up do you usually smoke your first cigarette?” was “0–5 minutes.”

Most callers (54%) had a high school education or less. However, Korean callers in general had more education. Only 6% of Koreans had less than a high school education, compared with 36% of Chinese and 40% of Vietnamese. Korean callers (43%) were more likely to have a bachelor’s degree than Chinese callers (22%) and Vietnamese callers (9%). Korean callers (6%) were also more likely to have a postgraduate degree than Chinese (3%) and Vietnamese callers (1%). Vietnamese (23%) were more likely to have some college, trade school, or an associate’s degree than Koreans (15%) or Chinese (10%).

Overall, 48% of callers had no health insurance. Vietnamese callers were more likely than Chinese or Koreans to have private health insurance (27% for Vietnamese; 21% for Chinese; 21% for Koreans) or public health insurance (36% for Vietnamese; 28% for Chinese; 24% for Koreans).

Ninety-six percent of all tobacco users were daily cigarette smokers. Slightly more Chinese (2%) than Koreans (0.4%) or Vietnamese (0.6%) were nondaily smokers. Although 36% of all smokers had their first cigarette in the first 5 minutes of waking, a smaller percentage of Vietnamese (30%) than Chinese (38%) or Korean (39%) callers reported doing so. Less than 1% of callers used noncigarette tobacco products, such as chew tobacco, cigars, or pipes.

### How people heard about the quitline and the services they received

Those who called for help with quitting were asked how they heard about ASQ. Nearly 54% of callers reported that they heard about ASQ via a newspaper or magazine; Chinese (58%) and Korean callers (57%) were more likely than Vietnamese (46%) to report this source (Table 2). Sixteen percent of callers heard about ASQ from another type of media; Vietnamese (24%) were more likely to report this than Korean (14%) or Chinese callers (10%). About 16% of callers heard about ASQ from friends or family; Chinese (13%) were less likely than Korean (19%) or Vietnamese callers (16%) to have heard about the quitline in this way. About eight percent of ASQ callers were referred by a health-care provider or a community organization. Chinese callers (13%) were the most likely to have heard about ASQ in this way, followed by Vietnamese (9%) and Korean callers (4%).

**Table 2:** How Tobacco Users Heard About the Asian Smokers’ Quitline, by Language, August 1, 2012–July 31, 2014

How did you hear about the quitline?	Chinese	Korean	Vietnamese	Total
	% (95% CI) [n]			% (n)
Newspaper/magazine	57.6 (55.3–60.0) [946]	57.2 (55.1–59.3) [1,211]	45.8 (43.4–48.1) [783]	53.8 (2,940)
Other media^a^	10.1 (8.6–11.5) [165]	14.3 (12.8–15.8) [302]	23.8 (21.8–25.8) [407]	16.0 (874)
Friend/family	12.8 (11.2–14.3) [210]	19.0 (17.4–20.7) [403]	16.4 (14.7–18.2) [281]	16.3 (894)
Health care/community organization^b^	12.7 (11.1–14.4) [209]	3.7 (2.9–4.5) [78]	9.0 (7.6–10.4) [154]	8.1 (441)
Don’t know/other	6.8 (5.5–8.0) [111]	5.8 (4.8–6.8) [123]	5.0 (4.0–6.1) [86]	5.9 (320)

Abbreviations: CI, confidence interval.

a Other media includes billboard/bus sign; mail; telephone directory; radio; television; Internet.

b Health care/community organization includes the following: clinic/doctor’s office; dentist/dental hygienist; health management organization/Medicaid/insurance; hospital; nonprofit organization; pharmacy; school; and the Special Supplemental Nutrition Program for Women, Infants, and Children (WIC).

Nearly all (99%) eligible (ie, not medically contraindicated) tobacco users received nicotine patches (Table 3). About 85% of tobacco users enrolled in counseling; Chinese callers (92%) were more likely than Korean (88%) or Vietnamese callers (76%) to enroll. Callers who enrolled in counseling and participated in at least 1 session attended a mean of 4 sessions, with no differences between language groups.

**Table 3:** Services Tobacco Users Received From the Asian Smokers’ Quitline, by Language, August 1, 2012–July 31, 2014

Services	Chinese	Korean	Vietnamese	Total
	% (95% CI) [n]			% (n)
Nicotine patches^a^	98.7 (98.1–99.3) [1,444]	99.4 (99.1–99.7) [2,004]	98.8 (98.2–99.3) [1,589]	99.0 (5,037)
Enrolled in counseling	91.5 (90.2–92.9) [1,502]	87.6 (86.2–89.0) [1,854]	76.4 (74.4–78.4) [1,307]	85.3 (4,663)
Counseling sessions,^b^ mean	4.2 (4.0–4.3) [1,316]	4.0 (3.9–4.1) [1,647]	4.1 (3.9–4.2) [1,141]	4.1 (4,104)

Abbreviations: CI, confidence interval.

a Includes all tobacco users eligible to receive medication (ie, not contraindicated), not just those enrolled in counseling.

b Mean number of counseling sessions is presented as number of unique telephone counseling sessions, not including the intake call. Sample comprises tobacco users who enrolled in counseling and completed at least 1 session.

## Discussion

During its first 2 years of operation, ASQ enrolled more than 5,400 Asian-language smokers from 48 states; 87% of these were male. More than half of all callers were aged 45 to 64, and education varied widely. Nearly all callers were foreign born, and very few were users of noncigarette tobacco products. Some variations by language among these demographic characteristics could be due to underlying population characteristics. For example, Korean callers generally had higher levels of education, which is consistent with how Koreans compare with Chinese and Vietnamese overall in the United States (16). Other differences are more difficult to interpret but may indicate varying levels of success in reaching subgroups of CKV speakers living in the United States. For example, despite advertisements featuring young adult male models, ASQ had the greatest success reaching middle-aged and older men.

Almost all eligible callers received nicotine replacement therapy. Cigarette smokers enrolled in counseling at a high rate, and those who participated in counseling completed an average of 4 sessions. In contrast, state quitlines in the United States that offer multiple counseling sessions generally report an average of 2.4 to 2.9 counseling sessions per caller (16), even though these quitlines may offer up to 5 calls (17). These findings are noteworthy for populations traditionally thought not to be receptive to behavioral counseling (7,11). ASQ strives to provide services in a culturally appropriate manner; for example, because Asian-language speakers tend to respect authority figures, ASQ coaches assume an authoritative tone when counseling callers (12). This study’s results are consistent with previous findings that Asian language smokers are willing to use services from a culturally tailored in-language quitline and actively engage in the counseling program once enrolled (7,8,11,12).

Nearly 54% of callers cited Asian-language newspapers and magazines, ASQ’s primary medium of promotion, as the way they heard about the service. In contrast, newspapers are a much less common referral source than television, word of mouth, or radio in studies of North American quitlines serving the general population (18). Other media used by partners to promote ASQ, including radio, television, and the Internet, were also cited with some regularity, showing that use of these media helped reach some who were not reached by newspaper advertising. About 16% of callers reported hearing about ASQ from friends and family, and 5% of all callers to ASQ were calling for someone else. This finding may suggest that Asian language-speaking smokers receive significant encouragement from friends and family to quit smoking. Thus, friends and family of smokers could represent a potential target for future ASQ promotions. Relatively few tobacco users (8%) cited health-care providers or community organizations as their sources of information about the ASQ, suggesting there may be opportunity for improvement and partnership building in these areas. However, nearly half of callers were uninsured, suggesting that many current callers may not have the support of a health-care provider in their cessation efforts.

Many states have relatively small populations of Asian language speakers (6) and most do not have tobacco use data on Asian-language speakers, making it difficult to offer and promote linguistically and culturally appropriate services locally to these populations. Thirty-four state quitlines offered services for CKV speakers in 2011 (19), but in every case except California, these were translation services. Although many racial/ethnic subgroups report satisfaction with state quitlines, surveys are generally completed in English and thus miss the experiences of those who use translation services (20). State health departments should consider whether their CKV populations would be better served by ASQ than by these translation services, which are cumbersome for callers and for quitlines, are not culturally tailored, and are less suitable for promotion to non-English populations.

Cigarette smoking prevalence varies among Asian subgroups, and the level of acculturation influences smoking rates and perceptions of the risks of tobacco use (4–6). Recent national data show wide variations across Asian American subgroups and sexes in current and ever-use of various tobacco products; however, these data are only available for English-speaking Asians. Nationally in 2010, there were 2.9 million CKV speakers with limited English fluency, with 38% of these in California, 14% in New York, and at least 2% in 10 other states (Florida, Georgia, Illinois, Maryland, Massachusetts, New Jersey, Pennsylvania, Texas, Virginia, and Washington) (21). States can review data posted on the ASQ website (11) to examine how many callers in each state have used ASQ services. Not all states (eg, Pennsylvania, Massachusetts) that rank in the top 10 for the size of CKV populations with limited English fluency (21) were in the top 10 for ASQ call volume. Despite 38% of the population of interest living in California, 47% of calls came from California. This difference may reflect the success of the California Smokers’ Helpline, which has promoted the Asian language services. In fact, California ran its own campaign promoting ASQ during the study period. The success of such efforts in California suggests that with adequate promotion and partnership building, use of ASQ in other states could also increase over time.

Referring more CKV speakers to ASQ can help reduce disparities in access to cessation services for these populations. With proper monitoring, evaluation, and promotional and outreach strategies, a single centralized quitline service for Asian language populations may be more efficient to operate and promote than multiple state and local services. Communities with sizable populations of CKV speakers can use the promotional resources available to them through CDC and Asian community partners such as the Asian Pacific Partners for Empowerment, Advocacy and Leadership (APPEAL). For example, the print ad for Asian language-speaking audiences that CDC produced as part of the Tips campaign (“Climb Out”) is available in CDC’s Media Campaign Resource Center (22), along with ASQ advertisements; states and nonprofit health partners can use these advertisements with no talent fees. Even if a state cannot afford paid media placement, they can use the free advertisements as public service announcements on state websites and social media pages. Innovative outreach strategies could also be employed and evaluated; one such effort in California trained bicultural, bilingual students to promote ASQ in Asian grocery stores. The educational initiative was well received and reached people with no previous awareness of quitline services (23). Other approaches targeting Asian communities, including smoke-free policies and other strategies to change social norms regarding the acceptability of tobacco use, could further encourage smoking cessation in these communities (24).

This study has at least 2 limitations. First, we were not able to calculate national reach because state-specific prevalence rates of CKV tobacco use outside of California are not available. In California, 1.2% of CKV-language smokers per year were reached by ASQ, which is comparable to the reach rates of US quitlines generally (24). ASQ reach could be approximated for other states by using an estimate of national tobacco use prevalence among CKV speakers (eg, 24.9% among foreign-born Asian men) (26) and the proportion of CKV speakers with limited English fluency in those states (using Census data). Second, we cannot generalize the results of this study to other Asian American or Native Hawaiian/Pacific Islander populations or to speakers of other languages. However, ASQ could be considered a potential model for other linguistic groups depending on assessment of their tobacco use profile, cultural/social context, and geographic dispersion.

Continuing to expand partnerships and promotion outside of California will be important to firmly establishing and sustaining ASQ as a national resource. To date, promotional efforts have focused primarily on running advertisements in Asian-language newspapers in urban areas with large CKV-speaking populations, and most callers reported hearing about ASQ from these advertisements. Identifying the most effective promotional methods and conducting an outcome evaluation to ensure that ASQ is achieving cessation outcomes comparable to those of previous published studies are important next steps. Communities with significant populations of CKV-language speakers can help reduce disparities in access to effective cessation services by promoting ASQ.
